# The dataset on the draft whole-genome sequences of two *Pseudomonas aeruginosa* strains isolated from urine samples of patients with urinary tract diseases

**DOI:** 10.1016/j.dib.2023.109704

**Published:** 2023-10-20

**Authors:** L.R. Valeeva, D.S. Pudova, N.N. Khabipova, L.H. Shigapova, E.I. Shagimardanova, A.M. Rogov, T.R. Tagirova, Z.G. Gimadeev, M.R. Sharipova

**Affiliations:** a Laboratory of Agrobioengineering, Institute of Fundamental Medicine and Biology, Kazan (Volga Region) Federal University, Parizhskoy Kommuny Str., 9, Kazan 420021, Russia; b ‘Regulatory genomics’ Research Center, Institute of Fundamental Medicine and Biology, Kazan (Volga Region) Federal University, Volkova Str. 18, Kazan 420012, Russia; c The Interdisciplinary Center “Analytical Microscopy”, Kazan (Volga Region) Federal University, Kazan (Volga Region) Federal University, Parizhskoy Kommuny Str., 9, Kazan 420021, Russia; d The Laboratory of clinical bacteriology, the Republican Clinical Hospital of the Republic of Tatarstan, Orenburgskiy trakt, 138, Kazan, 420064, Russia; e The Urological Department of the University Clinic in Kazan, Chekhova Str., 43, Kazan, 420043, Russia

**Keywords:** Pseudomonas aeruginosa, Uropathogen, Draft genome annotation, Virulence factors, Antimicrobial resistance

## Abstract

*Pseudomonas aeruginosa* is a widespread multidrug-resistant opportunistic human pathogen with an extremely high mortality rate that leads to urinary tract infection morbidities in particular. Variability and dynamics in genome features and ecological flexibility help these bacteria adapt to many environments and hosts and underlie their broad antibiotic resistance. Overall, studies aimed at obtaining a deeper understanding of the genome organization of UTI-associated *P. aeruginosa* strains are of high importance for sustainable health care worldwide. Herein, we report the draft assembly of entire genomes of two *P. aeruginosa* strains, PA18 and PA23, isolated from voided urine of patients with urinary tract diseases (hydronephrosis and urolithiasis, respectively) and determine the most important genetic features for pathogenesis and virulence. Whole-genome sequencing and annotation of genomes revealed high similarity between the two UTI strains along with differences in comparison with other uropathogenic *P. aeruginosa* and reference strains. The 6 981 635 bp and 6 948 153 bp draft genome sequences with GC contents of 65.9% and 65.8%, respectively, provide new insights into the virulence genetic factors and genes associated with antimicrobial resistance. The whole genome data of PA18 and PA23 have been deposited in the NCBI GenBank database (accession numbers JAQRBF000000000.1 and JAQRBG000000000.1, respectively).

Specifications Table

**Table:** 

Subject	Biology
Specific subject area	Genomics, Microbiology, Biomedicine
Type of data	Table Image Graph Figure
How the data were acquired	Datasets for the whole-genome sequences were acquired using Illumina MiSeq System and sequenced using a 2 × 300 bp paired-end sequencing kit. Data of genome annotation were obtained from the PGAP and RAST servers. Data on comparative analysis of genomes were acquired using Proteinortho v.6, BRIG, JSpeciesWV, MEGA v.11. Bacterial cells images were acquired via SEM (CarlZeiss, Inc., Germany). Strains identification were conducted using MALDI Biotyper based on Microflex/Autoflex MS (Bruker, Germany).
Data format	Raw Analyzed Filtered
Description of data collection	PA18 and PA23 strains were isolated from voided urine samples from patients with urinary tract infections (hydronephrosis and urolithiasis, respectively). Total DNA from bacterial cells was isolated using optimized phenol‒chloroform method. Sequencing libraries was prepared and assembled using NEBNext Ultra II DNA Library Prep kit for Illumina. Raw whole-genome sequence data were acquired on Illumina MiSeq System. For these sequences, NCBI Prokaryotic Genome Annotation Pipeline (PGAP), Rapid Annotations using Subsystems Technology (RAST) server were used for data collection.
Data source location	Institution: Institute of Fundamental Medicine and Biology, Kazan (Volga region) Federal University City/Town/Region: Kazan, Tatarstan Country: Russia
Data accessibility	Repository name: NCBI (National center for Biotechnology Information) GenBank Nucleotide database. Data identification number: Accession numbers JAQRBF000000000.1 and JAQRBG000000000.1 for *P. aeruginosa* PA18 and PA23, respectively, Direct URL to assembled data: For PA18 https://www.ncbi.nlm.nih.gov/nuccore/2442576946 For PA23 https://www.ncbi.nlm.nih.gov/nuccore/2442576961

## 1 Value of the Data

• The current data provide information on the whole-genome sequences of two *Pseudomonas aeruginosa* PA18 and PA23 isolates from the urine of patients with urinary tract diseases.
• Due to the extreme infectivity of pathogenic *P. aeruginosa* strains and high risks of associated mortality cases among patients [1,2], the provided data could contribute to new insights into genome flexibility, ecological and host adaptability, and genetic and molecular differences among *P. aeruginosa* strains. Consequently, the obtained data can help in the further development of more effective prevention strategies against *P. aeruginosa-*associated disease.
• The current data can be used by the biomedical community, including researchers in microbiology, genomics, molecular biology, and medicine, bacteriologists, medical personnel, and students of biological and medical academic institutions.
• The data could be used as additional information regarding pathogenic *P. aeruginosa* genomes for the further development of the *P. aeruginosa* databank and as the basis for molecular and evolutionary studies using reverse genetics. Draft genome sequences are available in GenBank and can be used to study antibiotic resistance and pathogenic factors. The data are also useful for the identification of new genetic determinants of *P. aeruginosa* pathogenicity that are extremely important in the improvement of new antibacterial compounds and UTI therapy.
• The whole-genome sequences of *P. aeruginosa* isolates associated with urinary tract diseases provide additional data for future molecular and genetic studies of urinary tract infection mechanisms, *P. aeruginosa* pathogenesis and antimicrobial resistance, along with host‒pathogen relation processes.
• The comparative analysis showed genomic similarities and differences among *Pseudomonas* strains associated with different diseases, and it also provides strict urinary tract disease-associated genomic features that could contribute to the development of new antimicrobial therapies.

## 2 Objective

The objective of this study was to provide whole-genome sequences and gene annotation information on two *Pseudomonas aeruginosa* clinical isolates, PA18 and PA23, from voided urine of patients with urinary tract diseases. *P. aeruginosa* has been implicated in a wide range of opportunistic infections, particularly urinary tract infections (UTIs) [3]. According to the latest data from global clinical studies, *P. aeruginosa* is among the five most mortiferous bacterial pathogens that most accurately demonstrates the importance of early and adequate treatment management [1]. However, the prevention and medical treatment of *P. aeruginosa*-associated infections is often difficult due to its multiple mechanisms of antimicrobial resistance [3]. Overall, the annotation of the whole-genome sequences of PA18 and PA23 isolated strains provides additional comparative information on the main genomic features of uropathogenic and related strains of *P. aeruginosa*. The obtained data on subsystem and gene annotation could contribute to the future molecular analysis of resistance mechanisms, pathogen‒host relations and UTI development.

## 3 Data Description

**Fig. 1:**
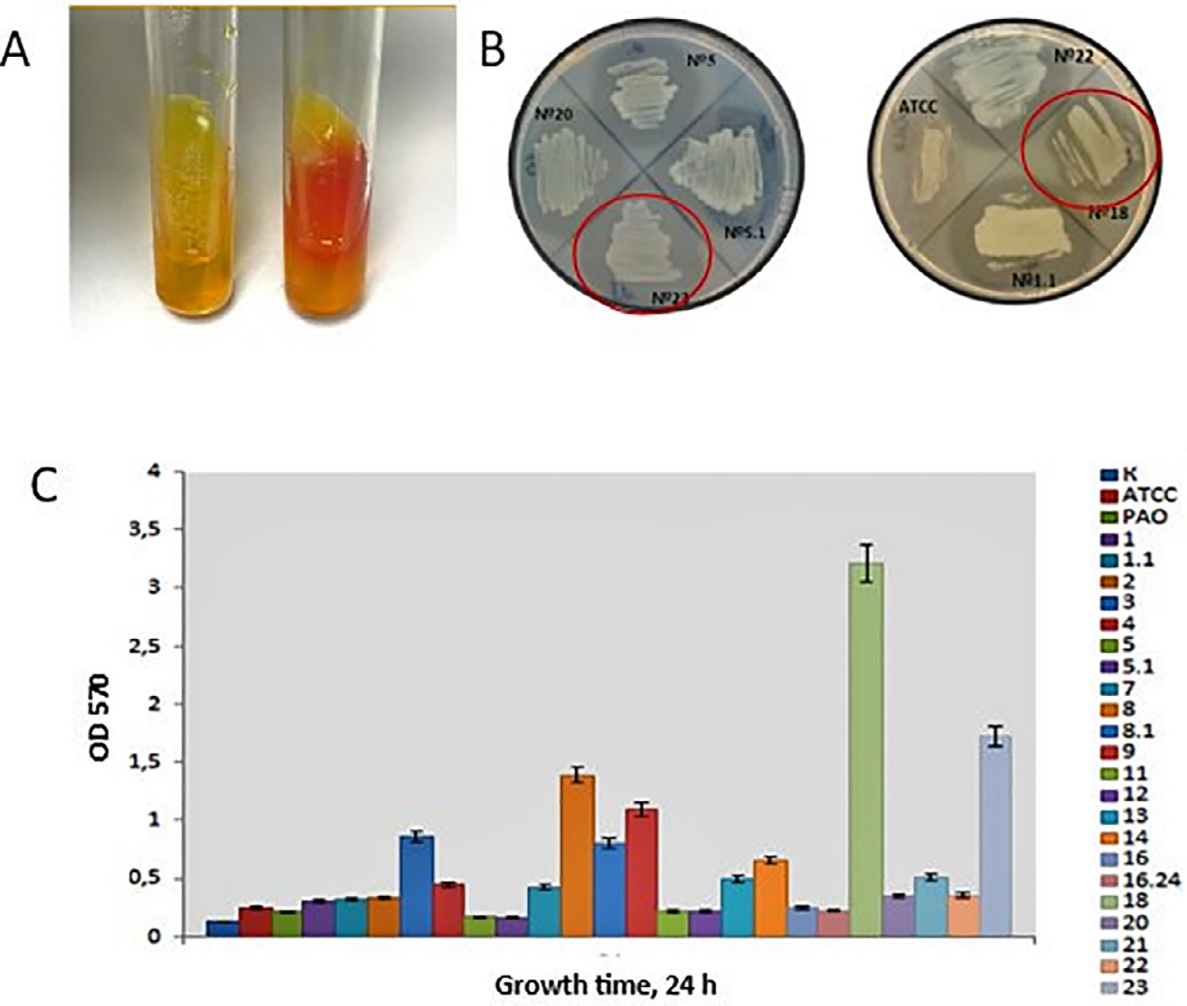
Characterization of activities of *P. aeruginosa* isolates. A. Urease activity. B. Protease activity. C. Biofilm-formation activity.

**Fig. 2:**
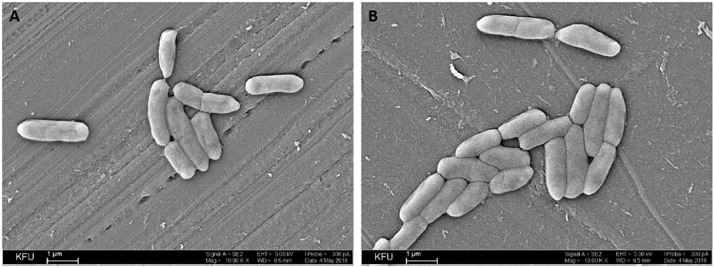
Morphology of *P. aeruginosa* isolates. Images were obtained via SEM. A. PA18. B. PA23. Magnification 10000х, scale bar 1 µm.

Two *P. aeruginosa* strains, PA18 and PA23, were isolated from the voided urine of patients with urinary tract diseases (hydronephrosis and urolithiasis, respectively) for the subsequent whole genome analysis. The urine sample inoculants were plated on elective medium (Columbia agar+5% sheep blood), and individual colonies were picked according to the typical *P. aeruginosa* colony morphology and pigmentation (blue‒green color). The taxonomy of selected strains was confirmed by molecular identification using MS (MALDI Biotyper). In total, twenty-three *P. aeruginosa* isolates were obtained; the following analysis of the biofilm formation ability of isolates and their protease and urease activity allowed us to identify the two most potentially active and virulent strains – *P. aeruginosa* PA18 and PA23 (Fig. 1). The morphology of bacterial cells was observed using scanning electron microscopy (SEM); both strains had rod-shaped morphology specific for the genus *Pseudomonas* (Fig. 2).

For whole-genome sequencing, total DNA was isolated from the 18 h cultures of the PA18 and PA23 strains grown under standard conditions. In this study, whole-genome sequence data of PA18 and PA23 were compared with genomes of related *P. aeruginosa* strains (Table 1).

**Table 1:** Bacterial strain genomes used in the study.

Bacterial strains	NCBI GeneBank	Source of isolation	Reference
*P. aeruginosa PA18*	JAQRBF000000000.1	Urine	this study
*P. aeruginosa PA23*	JAQRBG000000000.1	Urine	this study
*P. aeruginosa PAO1*	NZ_CP053028.1	Wound	[4]
*P. aeruginosa LESB58*	NC_011770.1	Lung sputum	[5]
*P. aeruginosa* 34Pae23	NZ_CP095772.2	Urine	[6]
*P. aeruginosa* 2810	NZ_RXCL00000000.1	Urine	[6]

The genome coverage of the P. aeruginosa PA18 and PA23 strains was 60.0 × and 90.0 ×, respectively. Based on the annotation provided by the Prokaryotic Genome Annotation Pipeline (PGAP), the draft genome sequence of PA18 constituted 69 contigs (>500 bp) and contained 6 981 635 bp with a G+C content of 65.9%. The whole genome of PA18 harbors 6614 coding sequences (CDSs), including 6532 protein-coding sequences, 71 RNA genes, 5 rRNA genes, 62 tRNA genes, and 4 ncRNA genes. The total number of pseudogenes in the PA18 genome was 82. On the other hand, the resulting assembly of the PA23 genome was composed of 174 contigs (>500 bp) and contained 6 948 153 bp with a G+C content of 65.8%. The PGAP server predicted 6579 CDSs within the PA23 genome with protein-coding sequences of 6475, total RNA coding genes of 68, 5 rRNA genes, 59 tRNA genes, and 4 ncRNA genes. The total number of pseudogenes in PA23 was 104 (Table 2).

**Table 2:** Comparative genomic features of isolated *P. aeruginosa* PA18 and PA23 and related strains.

Genome features	PA18	PA23	PAO1	LESB58	34Pae23	2810
Genome size (bp)	6,976,122	6,942,640	6,217,899	6601757	6,913,344	6,336,405
Chromosome size (bp)	6,976,122	6,942,640	6,217,899	6601757	6,870,367	6,336,405
Plasmids (size, bp)	N	N	N	N	42,977 (p34Pae23-KPC)	ND
G+C, chromosome (%)	65.9	65.8	66.5	66.3	66	66.4
Genes (total)	6685	6647	5745	6185	6519	5,943
CDS (total)	6614	6579	5671	6101	6437	5,877
Genes (coding)	6532	6475	5629	6042	6310	5,770
RNA genes	71	68	74	84	82	68
tRNA genes	62	59	61	67	65	57
ncRNA genes	4	4	4	5	5	3
rRNA genes	3, 1, 1 (5S, 16S, 23S)	3, 1, 1 (5S, 16S, 23S)	3, 3, 3 (5S, 16S, 23S)	4, 4, 4 (5S, 16S, 23S)	4, 4, 4 (5S, 16S, 23S)	8
Pseudogenes (total)	82	104	42	59	127	109
CRISPR arrays	4	N	N	1	N	1

**Fig. 3:**
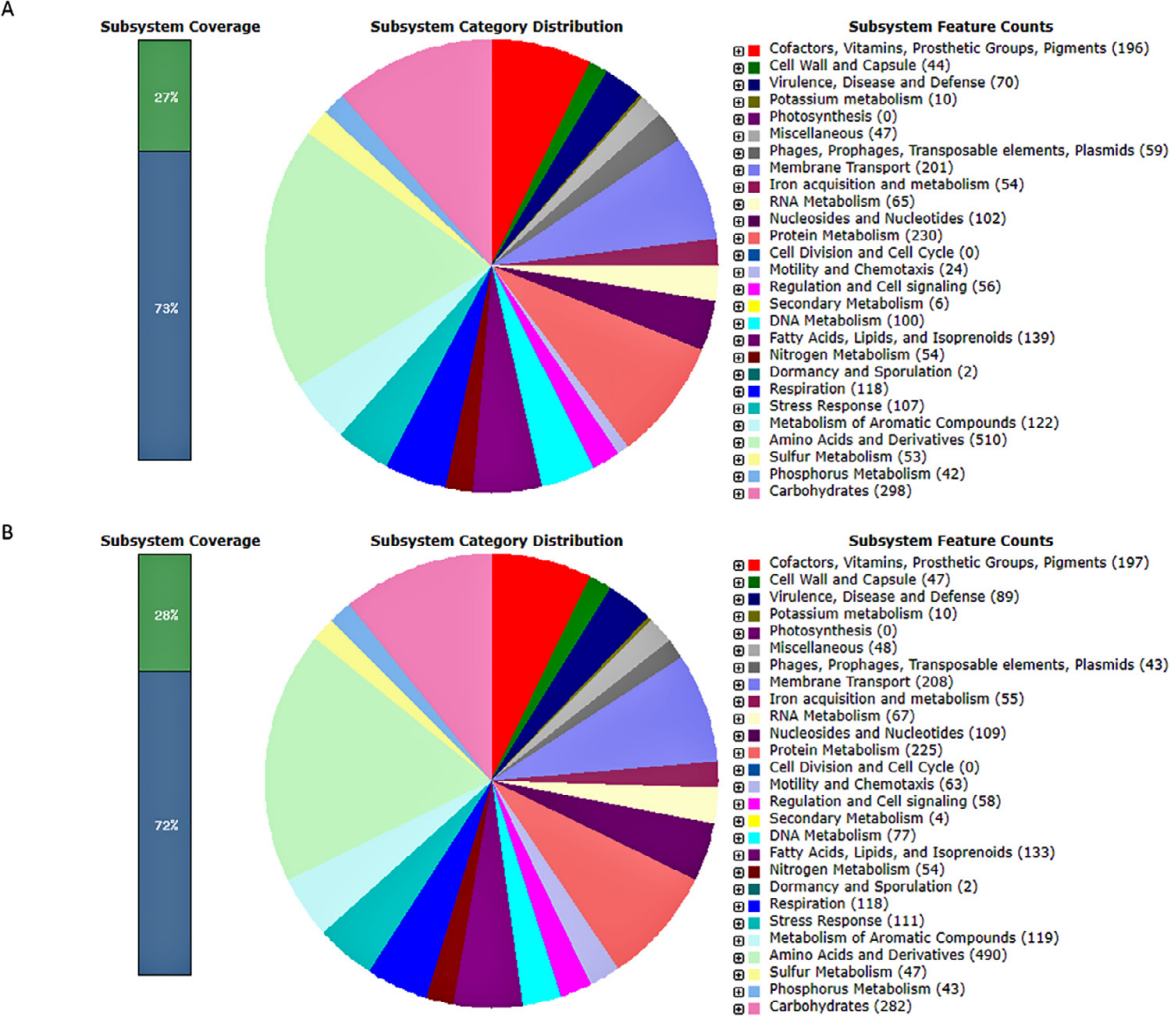
Subsystem distribution of the *Pseudomonas aeruginosa* genome based on the RAST annotation server. A. PA18 strain. B. PA23 strain. The bar shows the ratio of genes included in subsystems according to the SEED and not included. The pie chart displays the subsystems in each genome.

N – not identified; ND – no data;

PA18 genome annotation in RAST revealed 1866 CDSs (27%) as SEED subsystem features, and 5114 CDSs (73%) were identified as outside of the SEED subsystem; for the PA23 genome, 28% of CDSs were categorized as subsystem features, and 4881 CDSs (72%) were annotated as outside of the SEED subsystem (Fig. 3). Both the PA18 and PA23 strains possessed functional groups of genes that contain potential virulence factors, including motility, antimicrobial resistance, immune system evasion, hydrolytic enzymes, biofilm formation, QS, etc. However, there were some differences in virulence gene factor distribution within the two genomes (Table 3). Thus, PA18 demonstrated a broader spectrum of exotoxin genes in the T3SS system (*exoT, exoY,* and *exoS*), while PA23 had only two possible T3SS effectors (*exoY* and *exoT* genes). Such effector proteins injected directly into host cells through pathogen secretion system III suppress host cell defense and promote the infection process. Notably, it is likely that the *exoU* and *exoS* genes can be used as genetic markers of population diversification in *P. aeruginosa* [7].

**Table 3:** Virulence-associated subsystem distribution in the *P. aeruginosa* PA18 and PA23 genomes.

Subsystem	PA18	PA23
Urease subunits and urea decomposition	22	25
Dormancy and persistence	4	5
Siderophores	16	16
Membrane transport, protein secretion, type III	36	36
Membrane transport, protein secretion, type IV	34	32
Membrane transport, protein secretion, type II	15	15
Membrane transport, protein secretion, type VII	5	5
Motility, flagellar motility	21	56
Metalloendopeptidases (EC 3.4.24.)	5	6
cAMP signaling (including adenylate cyclase ExoY)	48	46
LysR-family proteins	33	33
Invasion and intracellular resistance	12	12
Resistance to antibiotics and toxic compounds	34	41

**Fig. 4:**
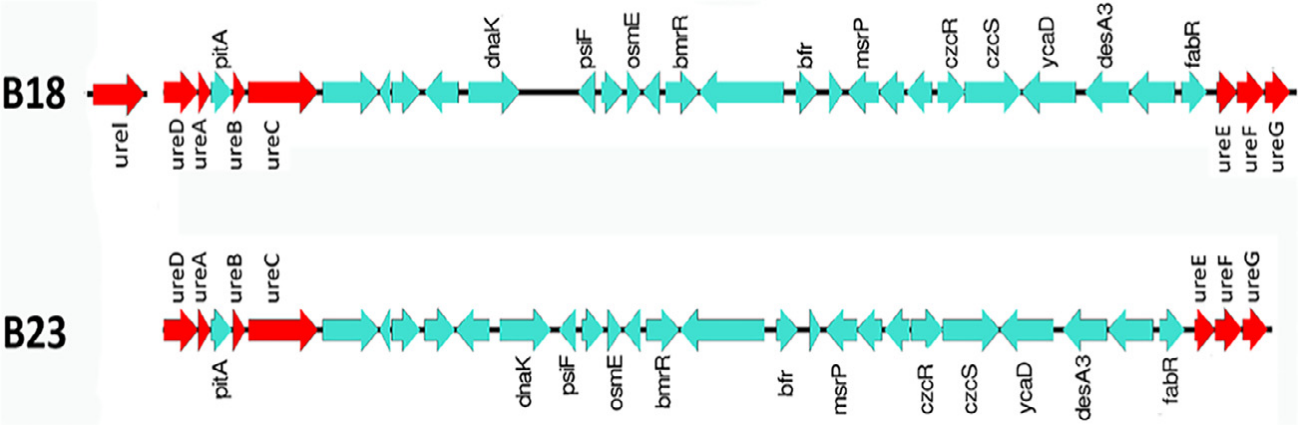
Urease coding genomic regions.

Urease genes were also found in both genomes, but the *ureI* urea importing channel gene was found only in the PA18 genome but not in the PA23 genome (Fig. 4). The number of genes involved in motility and synthesis of structures such as flagella and pili was two times higher in strain PA23 than in PA18 (56 vs. 21, respectively) (Table 3).

Other infection-associated genes were distributed equally in both genomes (Table 3). The RAST server predicted different types of secreted proteases involved in virulence, such as vibriolysin-like proteases and collagenases [8,9], in both the PA18 and PA23 genomes. Two crucial virulence factors, extracellular elastases AprA and LasB, which facilitate bacterial colonization and host tissue damage [10], were also represented in both genomes. Pyoverdine and pyochelin synthesis gene clusters (synthesis of siderophores – ferric ion chelators) were also predicted in both strains. Essential components of membrane transport systems involved in the secretion of bacterial exotoxins, exoenzymes, siderophores, and pilus and flagella structural proteins were also represented in the PA18 and PA23 genomes.

**Fig. 5:**
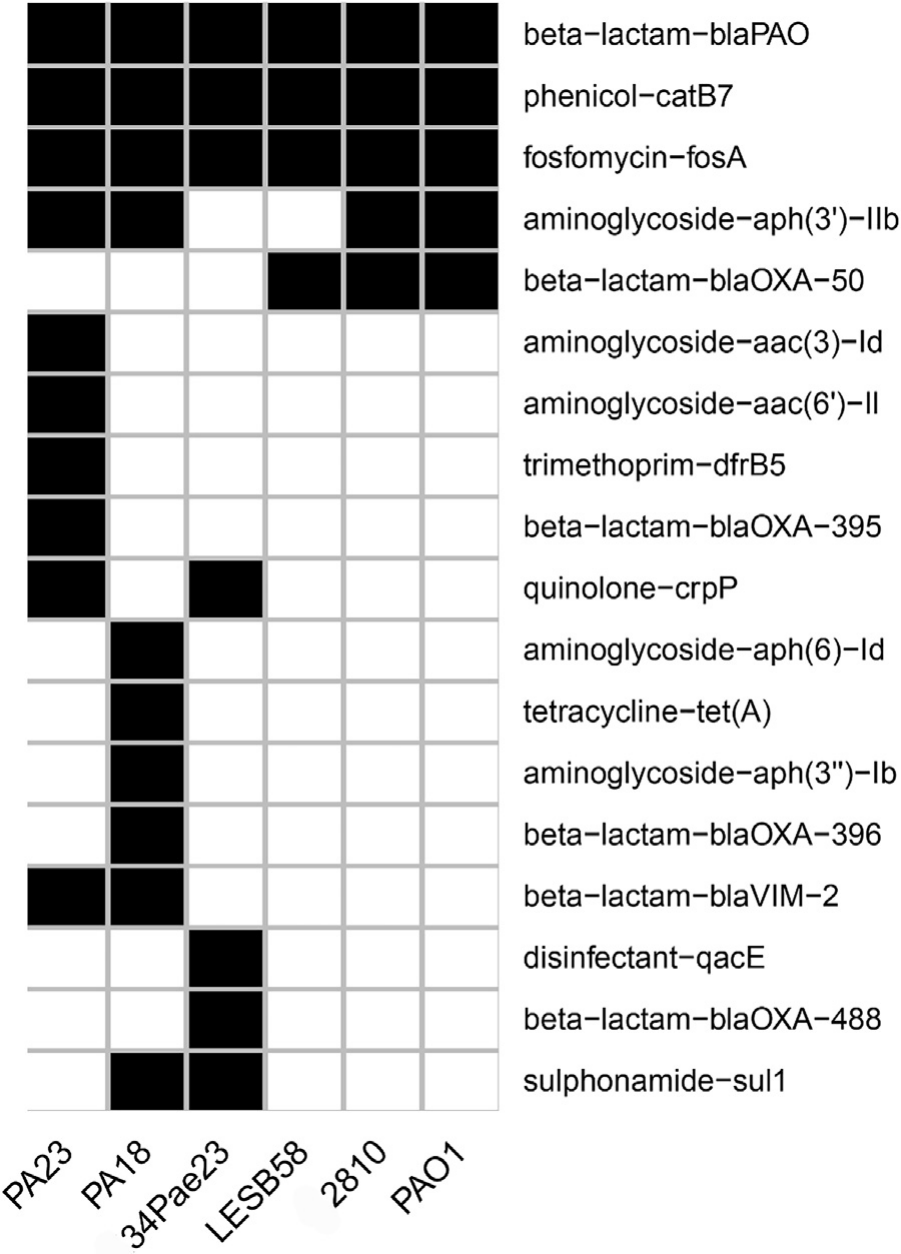
Comparative chart of antibiotic resistance gene factors in the PA18 and PA23 genomes and related *P. aeruginosa* genomes (PAO1 (reference), LESB58, 34 Pae23, 2810).

Comparative analysis of antimicrobial resistance genes revealed that both the PA18 and PA23 strains harbored 10 antibiotic resistance (ABR) genes, while the PAO1 and 2810 strains had only 5 ABR genes and LESB58 strain had only 4 genes (Fig. 5). Seven ABR genes were found in the *P. aeruginosa* 34PAe23 genome. Antimicrobial resistance profiling of PA18 was conducted using ResFinder and RAST servers, predicted genes encoding β-lactamase genes and associated proteins (12 genes), 3’-, 3’’- and 6’-aminoglycoside phosphotransferases (4 genes, aph(3’)-IIb, aph(3’’)-Ib, aac(6’)-II), coding sequences conferring resistance to fluoroquinolone (DNA gyrase A and B subunits), chloramphenicol acetyltransferase gene (amphenicol-cat B7), and fosfomycin resistance protein (FosA). Interestingly, tetracycline resistance was provided by different mechanisms: one tetracycline resistance efflux pump and target modification (translation elongation Factor G resistant to tetracycline). The analysis of antibiotic resistance-associated genes of strain PA23 revealed 11 genes of β-lactamase genes and associated proteins, 4 genes of 3’-, 3’’- and 6’-aminoglycoside phosphotransferases, genes of gyrase (subunit A and B) involved in fluoroquinolone resistance, chloramphenicol O-acetyltransferase gene (EC 2.3.1.28), and fosfomycin resistance protein (FosA). Tetracyclin resistance of PA23, similar to PA18, was represented by efflux systems and target modification (translation elongation Factor G resistant to tetracyclin). Compared to the PAO1 reference genome, the genomes of the two urea isolates, PA18 and PA23, are more armored with genes of β-lactamases (11 and 12 genes in PA18 and PA23 vs. 2 genes in PAO1). Moreover, aminoglycoside phosphotransferase genes were more abundant in isolate genomes than in PAO1 (4 genes vs. 1 gene, respectively). Additionally, seventeen and twenty predicted regions encoding multidrug efflux systems were found in the PA18 and PA23 genomes, respectively.

Prophages play a considerable role in the short-term genome evolution of bacteria, particularly opportunistic pathogens such as *P. aeruginosa*, contributing to virulence and pathogenicity, including antibiotic resistance, motility, biofilm formation and immune system suppression [11]. Prophage genes were identified in both the PA18 and PA23 genomes (Tables 4, 5). Nevertheless, the analysis of the PA18 genome revealed more predicted prophages than in PA23 (10 and 5, respectively). Seven prophages in the PA18 genome were classified as intact and 3 as questionable, while in the PA23 genome, 3 intact and 2 questionable prophages were identified (Tables 4, 5). A common *Pseudomonas* sp. prophage PHAGE Pseudo F10 NC 007805 [12] was detected in two genome regions of P18 (48-28747 bp and 4808244-4859138 bp). However, it was determined to be intact only in the first region (score 120), while the second region was questionable (score 90). A similar intact prophage (Score 150) was also found in the PA23 genome in the region of 873891-913141 bp. Another prophage found in both of the genomes was a questionable sequence of PHAGE Pseudo Pf1 NC_001331 that is also often identified in *P. aeruginosa* genomes [13]. In addition to *Pseudomonas-*related prophage sequences of prophages specific for other bacteria, 2 intact prophages (PHAGE Escher vB EcoM ep3 NC_025430, PHAGE Burkho AH2 NC_018283) and 1 questionable PHAGE Entero H66 NC 042342 were found in the PA18 genome, and vB EcoM -ep3 phage was previously found in the genome of multidrug-resistant *E. coli* clinical isolates [14]. It was shown that Lysep3 lysin from this phage was 58% similar to the *Pseudomonas* phage PppW-3 lysin [14]. A questionable phage, Burkho AH2 NC_018283, detected in the PA18 genome was initially found in *Burkholderia cepacia,* which was previously known as a member of the *Pseudomonas* genus [15]. A questionable PHAGE Entero N15 NC_001901 was also detected in the PA18 genome. Interestingly, N15 is a lambdoid linear plasmid prophage that was originally identified in *E. coli* [16].

**Table 4:** Prophage-associated sequences in the *P. aeruginosa* PA18 genome.

	Size, kb	Score	Protein number	Genome localization, bp	Predicted Prophage	GC, %
1	28.7	120	45	48-28747	PHAGE_Pseudo_F10_NC_007805(16)	62.57
2	44.1	137	49	738240-782396	PHAGE_Pseudo_phi3_NC_030940(33)	64.66
3	18.4	80	25	812005-830479	PHAGE_Pseudo_Dobby_NC_048109(6)	65.30
4	16.2	96	15	900819-917079	PHAGE_Pseudo_Pf1_NC_001331(9)	60.78
5	55.3	150	53	1260928-1316282	PHAGE_Escher_vB_EcoM_ep3_NC_025430(7)	63.60
6	65	150	82	1762786-1827880	PHAGE_Burkho_AH2_NC_018283(16)	60.88
7	62.8	150	81	2307130-2369951	PHAGE_Pseudo_PMG1_NC_016765(37)	59.14
8	9.9	70	15	3572446-3582427	PHAGE_Entero_N15_NC_001901(3)	60.31
9	66.7	130	68	3639663-3706454	PHAGE_Pseudo_H66_NC_042342(57)	63,27
10	50.8	90	57	4808244-4859138	PHAGE_Pseudo_F10_NC_007805(36)	59.68

**Table 5:** Prophage-associated sequences in the *P. aeruginosa* PA23 genome.

No	Size, kb	Score	Protein number	Genome localization, bp	Predicted Prophage	GC, %
1	18.4	80	25	614079-632554	PHAGE_Pseudo_phiCTX_NC_003278(6)	65.29
2	18.8	75	19	708105-726994	PHAGE_Pseudo_Pf1_NC_001331(7)	53.73
3	39.2	150	57	873891-913141	PHAGE_Pseudo_F10_NC_007805(18)	62.15
4	41.4	95	51	2224902-2266316	PHAGE_Pseudo_JBD67_NC_042135(33)	61.59
5	36.1	100	45	6910520-6946657	PHAGE_Pseudo_JBD44_NC_030929(15)	59.62

**Fig. 6:**
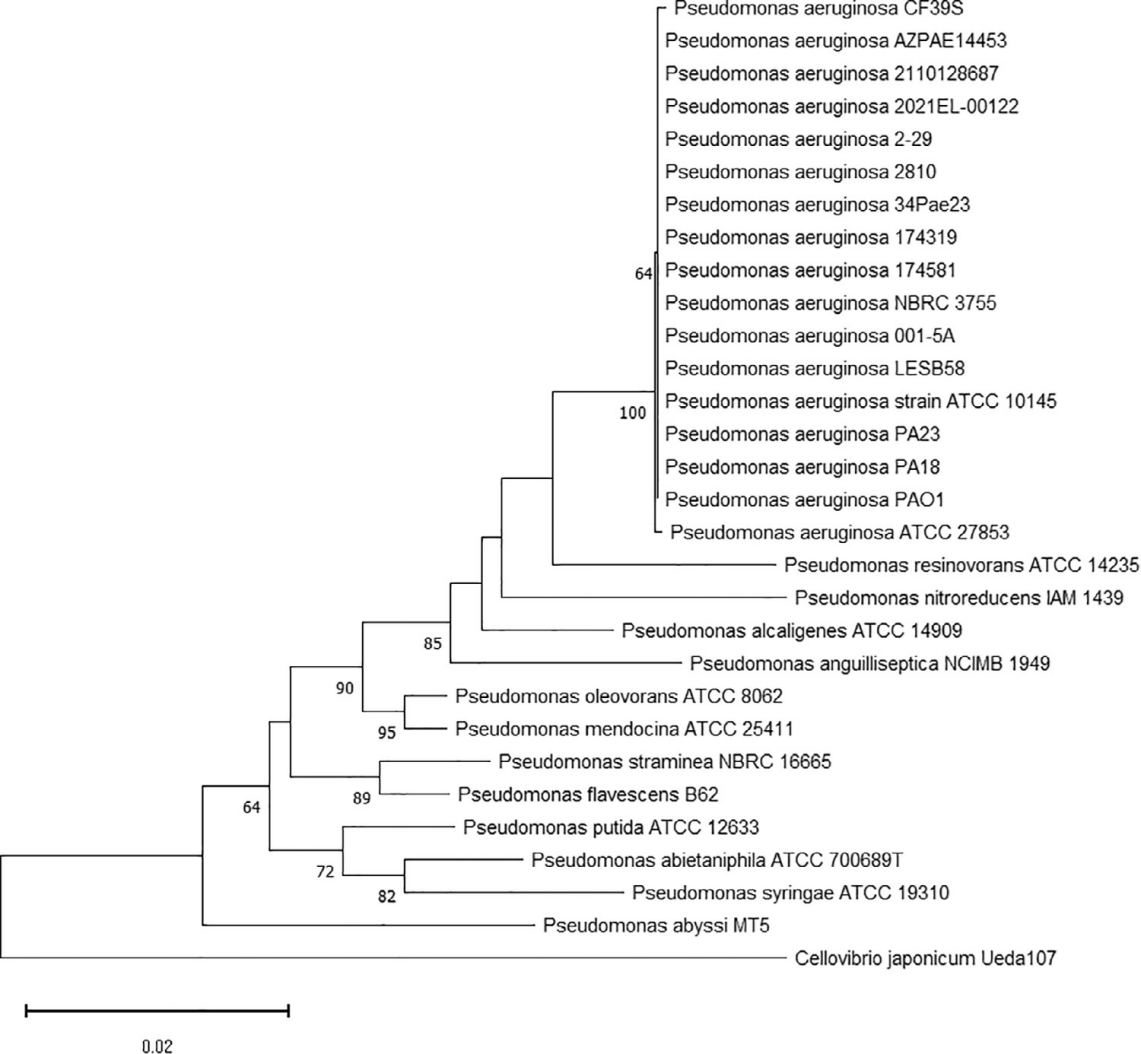
Phylogenetic tree based on 16S rRNA gene sequences of PA18 and PA23 and related *Pseudomonas* species. Analysis was performed in MEGA11 by using the neighbor-joining method. The percentage of replicate trees in which the associated taxa clustered together in the bootstrap test (1000 replicates) are shown next to the branches. *Cellovibrio japonicum* Ueda107 was used as an outgroup species.

According to current genomic data, the phylogenetic positions of isolated strains were reconstructed using comparative analysis of PA18 and PA23 strain 16S rRNA gene sequences with their closely related *Pseudomonas* homologs (Fig. 6). Both *P. aeruginosa* PA18 and PA23 were closely related to other *P. aeruginosa* species isolated from urine, blood, and lung samples associated with UTI, sepsis, and cystic fibrosis; on the other hand, PA18, PA23, and other *P. aeruginosa* strains were phylogenetically farther from *P. aeruginosa* ATCC 27853 (strain Boston #41501 isolated from blood culture), which could be explained by the diversification within *P. aeruginosa* species.

**Fig. 7:**
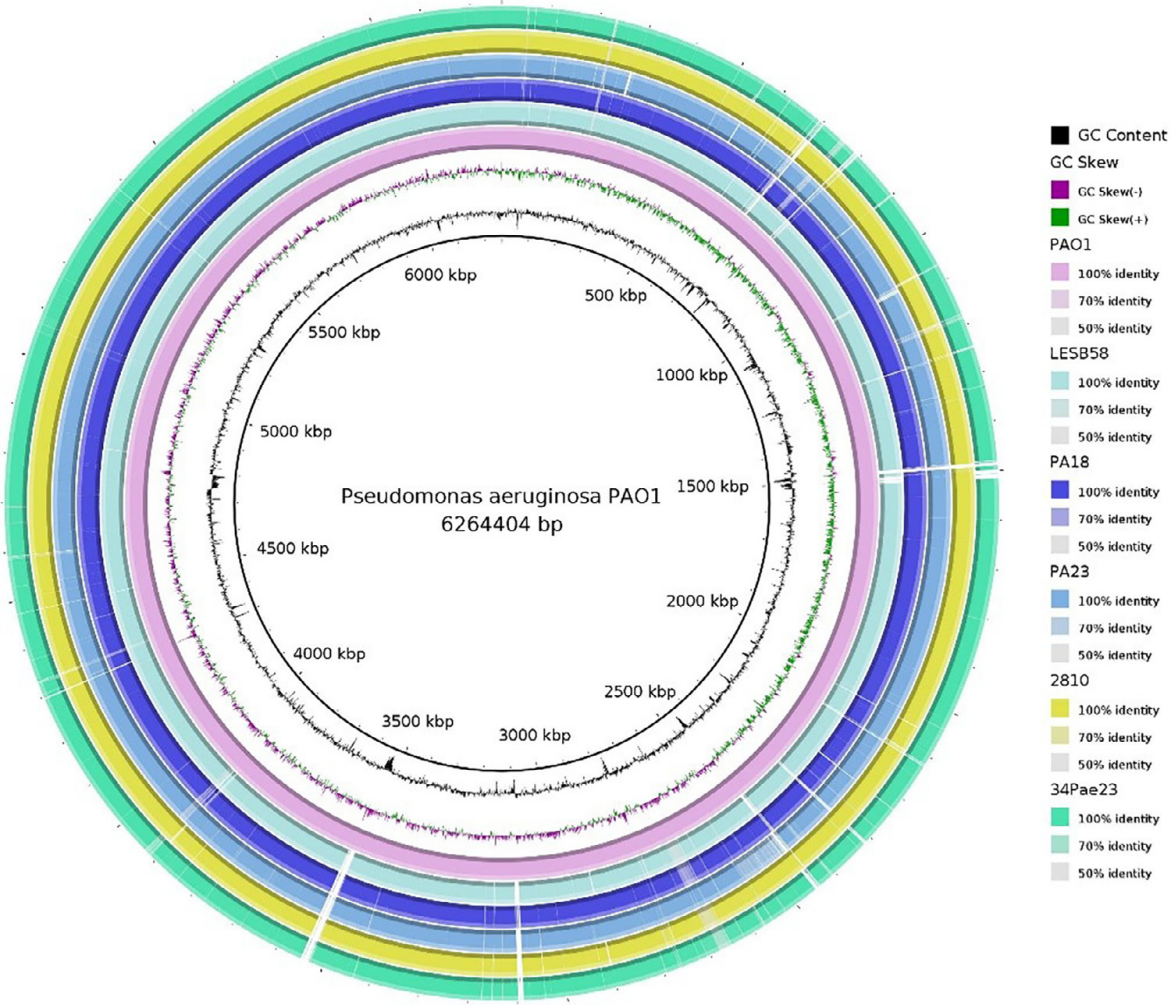
Blast Ring Image Generator (BRIG) plot showing a whole genome comparison. The figure shows BLAST comparisons of strains isolated in this work, PA18, PA23, and related *P. aeruginosa* strains PAO1, LESB58, 34 Pae23, and 2810.

The whole-genome sequence comparison of PA18 and PA23 with both the reference genome PAO1 and three genomes of pathogenic *P. aeruginosa* strains (Table 1) revealed an ANI score >95% (Table 6). Considering this fact, *P. aeruginosa* genomes remain relatively permanent despite the ecological variety of bacteria, showing the absence of strong host specificity of *P. aeruginosa* that could explain their adaptability and widespread distribution. PA18 had a closer position to PAO1, LESB58, and 2810 (ANI scores of 99.44%, 99.40%, and 99.46%), while PA23 was closer to the 34 Pae23 strain (ANI score of 99.26%) (Table 6). Visualization of genome comparisons between closely related *P. aeruginosa* strains (PA18, PA23, PAO1, 34PAe23, and 2810) was provided by BRIG (Fig. 7).

**Table 6:** ANIm (average nucleotides aligned based on MUMmer algorithm) calculation for *P. aeruginosa* PA18, PA23, PAO1, 34 Pao23, and 2810 strains.

	PA18	PA23	PAO1	LESB58	34Pae23	2810
PA18		98.84	99.44	99.40	98.83	99.46
PA23	98.83		98.96	98.84	99.26	98.94
PAO1	99.44	98.96		99.47	98.94	99.44
LESB58	99.40	98.84	99.47		98.87	99.47
34Pae23	98.83	99.26	98.84	98.87		98.94
AZPAE14453	99.45	98.95	99.44	99.47	98.94

**Fig. 8:**
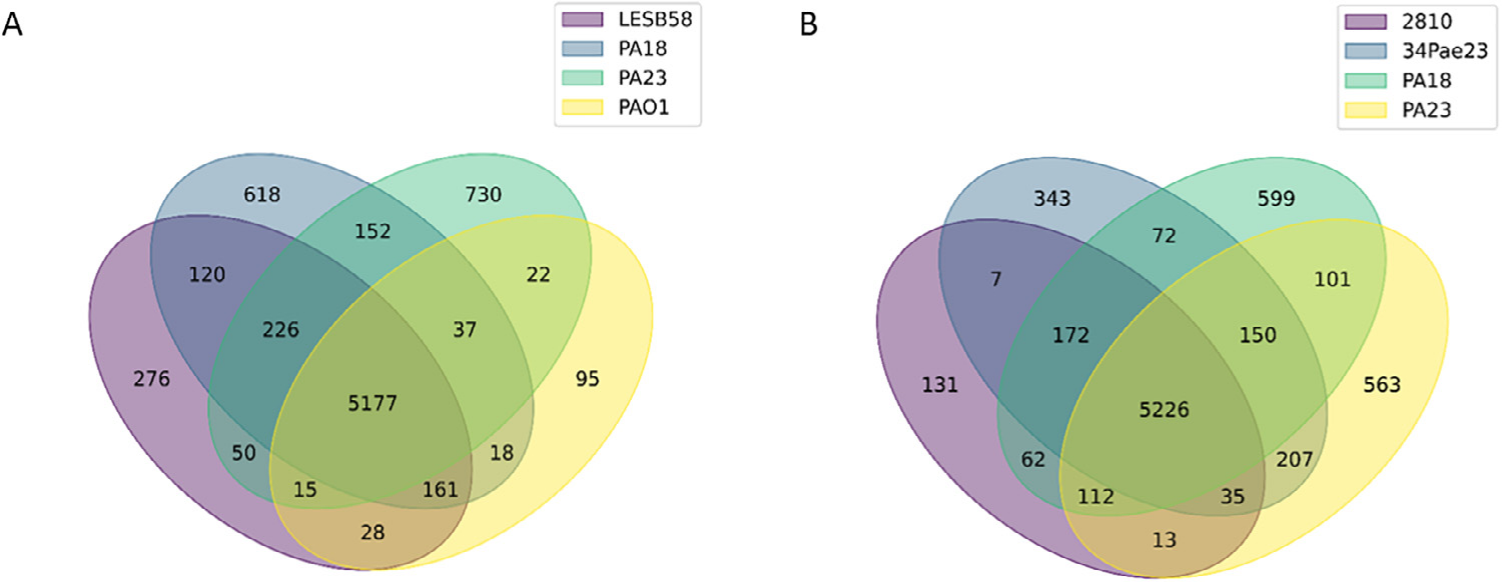
Analysis of orthologous gene clusters using ProteinOrtho. A. Venn diagram presentation for orthologous genes of isolated PA18 and PA23 strains and reference PAO1 and LESB68 genomes. B. Venn diagram showing the number of common and unique genes among isolated PA18 and PA23 strains and related uropathogenic 34 Pao23 and 2810 genomes. The numbers indicate unique and shared genes among the analyzed genomes.

Comparison of the PA18 and PA23 genomes with genomes of reference strains PAO1 and LESB58 by Proteinortho v.6 software predicted 5177 common genes, where PA18 consisted of 618 unique genes and PA23 included 730 unique genes almost associated with prophage or hypothetical genes containing genome regions (Fig. 8A). Moreover, antibiotic resistance-associated genes such as tetracycline efflux MFS transporter Tet(A) (protein accession number MDC8989344.1), tetracycline resistance transcriptional repressor TetR(A) (MDC8989345.1), sulfonamide-resistant dihydropteroate synthase Sul1 (protein accession number MDC8989358.1), aminoglycoside O-phosphotransferase APH(6)-Id (protein accession number MDC8989361.1), and aminoglycoside O-phosphotransferase APH(3′')-Ib (protein accession number MDC8989362.1) were also unique to PA18 compared to PAO1. Genes encoding the aminoglycoside N-acetyltransferase AAC(3)-Id (MDC9031207.1), trimethoprim-resistant dihydrofolate reductase DfrB5 (MDC9031208.1), aminoglycoside N-acetyltransferase AAC(6′)-Il (MDC9031210.1), and multidrug efflux RND transporter permease subunit (MDC9031195.1) were unique to PA23 in comparison to PAO1 and PA18.

On the one hand, comparison of the PA18 and PA23 genomes with uropathogenic *P. aeruginosa* 34 Pae23 and 2810 strains predicted more common genes (5226) with fewer unique genes (343 and 599 for PA18 and PA23, respectively) (Fig. 8B). Regardless, unique CRISPR system-associated genes remained unique for PA18 in comparison with the 2810 strain. Additionally, it was shown that only 4 of 5 antibiotic resistance-associated genes listed above were unique for PA18: sulfonamide-resistant dihydropteroate synthase Sul1 (protein accession number MDC8989358.1) was also found in other uropathogenic strains. Genes encoding multidrug efflux RND transporter permease subunit (MDC9031195.1) and trimethoprim-resistant dihydrofolate reductase DfrB5 (MDC9031208.1) were unique for PA23 among comparing uropathogenic strains.

**Fig. 9:**
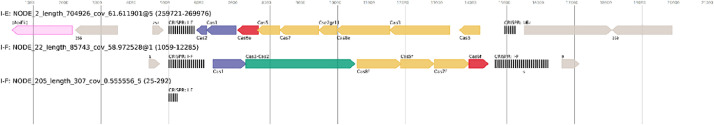
CRISPR system-associated genes and CRISPR arrays in the *P. aeruginosa* PA18 genome.

Among PA18 unique genes, a total of 13 CRISPR system protein-encoding genes were found, including I-E and I-F CRISPR types, which were not found in the PA23 genome (Table 7, Fig. 9).

**Table 7:** CRISPR system-associated proteins found in *P. aeruginosa* PA18.

	Protein accession number	Protein name
Type I-E	MDC8987161.1	type I-E CRISPR-associated endoribonuclease Cas2e
	MDC8987162.1	type I-E CRISPR-associated endonuclease Cas1e
	MDC8987163.1	type I-E CRISPR-associated protein Cas6/Cse3/CasE
	MDC8987164.1	type I-E CRISPR-associated protein Cas5/CasD
	MDC8987165.1	type I-E CRISPR-associated protein Cas7/Cse4/CasC
	MDC8987166.1	type I-E CRISPR-associated protein Cse2/CasB
	MDC8987167.1	type I-E CRISPR-associated protein Cse1/CasA
Type I-F	MDC8991778.1	type I-F CRISPR-associated endonuclease Cas1f
	MDC8991779.1	type I-F CRISPR-associated helicase Cas3f
	MDC8991780.1	type I-F CRISPR-associated protein Csy1
	MDC8991781.1	type I-F CRISPR-associated protein Csy2
	MDC8991782.1	type I-F CRISPR-associated protein Csy3
	MDC8991783.1	type I-F CRISPR-associated endoribonuclease Cas6/Csy4

Despite the fact that many *P. aeruginosa* strains are dangerous human pathogens, this microorganism is also considered a producer of antibacterial compounds due to its high adaptivity, metabolism variability, and competition capacity. It was shown that *P. aeruginosa* utilizes various proteins and secondary metabolites to provide advantages in interspecies competition for survival [2]. For example, the effector protein Tse2-6 (phospholipase D) secreted by the T6SS secretion system is injected by *P. aeruginosa* into the periplasm of other bacteria to damage peptidoglycan and kill them, which helps in competition for colonization of the environment [2]; on the other hand, *P. aeruginosa* can also produce various bacteriocins and secondary organic metabolites, which might be used as effective antibacterial compounds. It was shown that *P. aeruginosa* UICC B-40 produced the (2*E*,5*E*)-phenyltetradeca-2,5-dienoate compound (molecular weight 300 g/mol), composed of a phenolic ester, fatty acid and long chain of aliphatic group structures, which had antibacterial effects against another human pathogen, gram-positive *Staphylococcus aureus* [17]. Another strain, *P. aeruginosa* LV, is actively studied for its antibacterial activity due to the production of organocopper compounds [18]. It was also found that bacteriocins, particularly R-pyocins, produced by *P. aeruginosa* had antibacterial effects against biofilms and could be used as antibacterial agents [19]. Fifty-three proteins belonging to the T6SS system and its effectors, including Tse2, Tse3, and Tse5, were found in both the PA18 and PA23 genomes. However, the Tse7 effector DNase and PldB phospholipase D were found only in PA18, while the peptidoglycanhydrolase Tse1 and TseT effector genes were unique to the PA23 genome. Seven genes associated with bacteriocin synthesis were found in the PA18 genome, including lipid II-degrading bacteriocin, S-type pyocin domain-containing protein, and colicin immunity domain-containing protein. The PA23 genome also contained 7 genes associated with bacteriocin production; however, in contrast to PA18, the genome of PA23 contained bacteriocin immunity protein and pyocin killing protein but not colicins. In that case, the analysis of *P. aeruginosa* genomes can also provide new information on the distribution of genes involved in the production of antagonistic compounds, which could help not only in understanding pathogen adaptation to multispecies environments but also in the development of new antibacterial compounds.

Overall, the comparative whole-genome analysis of the uropathogenic *P. aeruginosa* isolated in this work with other uropathogens and other human pathogens revealed both high species-specific genetic feature distribution in the genomes and higher similarities between uropathogenic genomes. The current study might help to identify genetic features specifically associated with uropathogenic genomes of *P. aeruginosa* involved in the UTI process.

## 4 Experimental Design, Materials, and Methods

### 4.1 Collection of *Pseudomonas Aeruginosa* Isolates

Urine samples were obtained from patients with urinary tract infections at the Urological Department of the University Clinic in Kazan, the Republic of Tatarstan, Russia. Samples were obtained according to the ethical approval of the Biomedicine Ethics Expert Committee of Kazan Federal University (No. 218, 11.15.2012). Urine samples were immediately used for bacterial inoculation on elective Columbia agar medium containing 5% defibrinated sheep blood in Petri dishes. Inoculates were incubated for 24 h at 37°C. Single colonies were transferred into new plates with meat-infusion agar medium and grown under the same standard conditions. *P. aeruginosa* candidate strains were selected according to blue‒green colony pigmentation. Identification of the bacterial strains was carried out using unique protein fingerprint profiling by MALDI Biotyper based on Microflex/Autoflex mass spectrometers (Bruker, Germany). A single colony was picked from a fresh bacterial culture, stirred on an MTP target and dried at room temperature. One microliter of 2,5-DHB (2,5-dihydroxybenzoic acid) matrix solution was dropped onto the sample and dried (treated two times). Samples were identified according to the spectrum of ribosomal proteins by MALDI Biotyper mass spectrometry based on the MALDI databases. The spectra were processed using Bruker DataAnalysis, Version 1.6 software.

### 4.2 Scanning Electron Microscopy

Microscopy was performed in the Interdisciplinary Center “Analytical Microscopy” at KFU. The samples were analyzed using a scanning electron microscope (CarlZeiss, Inc., Germany) in high vacuum mode at 2 kV. Sample preparation was carried out according to the standard procedure [20].

### 4.3 Total DNA Extraction and Quality Control, Library Preparation and Sequencing

Total DNA extraction was performed by an optimized phenol method. DNA quality was analyzed by electrophoresis; DNA quantity was measured by a Qubit 4 spectrophotometer (Thermo Scientific). For DNA libraries for whole-genome sequencing, total DNA samples were previously fragmented by sonication in a Q800R2 Sonicator (Qsonica), followed by preparation using the NEBNext Ultra DNA Library Prep Kit for Illumina (New England Biolabs) according to the manufacturers' instructions. The quality of the DNA library was controlled using a 2100 Bioanalyzer (Agilent) and a High Sensitivity DNA Kit (Agilent). Sequencing was performed using a MiSeq Reagent Kit v2PE 00 cycles (Illumina) on a high-throughput Illumina MiSeq platform (Illumina) at the ‘Regulatory genomics’ Research Center, Kazan (Volga Region) Federal University, (Kazan, Russia).

### 4.4 Genome Assembly

The obtained sequence read quality was determined using FastQC version 0.11.7 (https://www.bioinformatics.babraham.ac.uk/projects/fastqc). Reads with a quality value of Q<20 were excluded from further analysis by Trimmomatic version 0.36. *De novo* genome assembly was conducted using SPAdes version 3.12. The quality of assemblies was assessed using metrics implemented in QUAST [21].

### 4.5 Whole-Genome Annotation

Genome annotation was carried out using the NCBI Prokaryotic Genomes Annotation Pipeline. Draft genome contigs were aligned with reference genomes (*P. aeruginosa* PAO1, RefSeq accession number NZ_CP053028.1; *P. aeruginosa* LESB58, RefSeq accession38. number NC_011770.1; *P. aeruginosa* 34 Pao23, RefSeq accession number NZ_CP095772.2; *P. aeruginosa* 2810, RefSeq accession number NZ_RXCL00000000.1) (Table 1). Functional annotation was conducted using the RAST server. The average nucleotide identity analysis used to select the closely related reference strain was carried out by JSpeciesWS using the MUMmer algorithm (https://jspecies.ribohost.com/jspeciesws/) [22]. Antimicrobial resistance genes harbored within the genomes were identified through functional annotation generated from the ResFinder v. 4.1 platform (https://cge.food.dtu.dk/services/ResFinder/), and the related heatmap was generated in RStudio using the “gplots” package. CRISPR systems were analyzed, and a scheme was generated by (https://crisprcastyper.crispr.dk/). Prophage sequences were analyzed by PHASTER (https://phaster.ca/). PHASTER classified all prophage sequences as intact (score > 90), questionable (score 70-90), or incomplete (score < 70) based on the presence of specific phage-related products of genes (such as ‘capsid’, ‘head’, ‘integrase’, ‘plate’, ‘tail’, ‘transposase’, ‘portal’, and other), size of the region and amount of proteins. Multiple genome alignment was preceded using Blast Ring Image Generator (BRIG) (https://brig.sourceforge.net/) [23]. Visualization of urease coding genomic regions was carried out by the Easyfig v. 2.2.2 program [24]. Proteinortho v. 6 (http://gitlab.com/paulklemm_PHD/proteinortho) was used for identification of orthologous protein groups in comparing genomes followed by visualization on a Venn diagram using Python 3. The area of intersection in the Venn diagram for the studied strains indicates the number of orthologous groups containing proteins from the genomes of these strains. Alignment of 16S rRNA gene sequences was carried out by ClustalW. There were a total of 1408 positions in the final dataset. A phylogenetic tree was generated by MEGA 11 (https://www.megasoftware.net/) [25] using the neighbor-joining method. The evolutionary distances were computed using the maximum composite likelihood method. The percentage of replicate trees was obtained from 1000 bootstrap replicates.

## Ethics Statement

The study was approved by the Local Ethics Committee of Federal State Autonomous Educational Institution of Higher Education “Kazan (Volga Region) Federal University”, under the institutional and international ethical guidelines, protocol No 41, May 23, 2023.

## CRediT authorship contribution statement

**L.R. Valeeva:** Software, Writing – original draft, Writing – review & editing. **D.S. Pudova:** Software, Formal analysis. **N.N. Khabipova:** Investigation, Writing – original draft. **L.H. Shigapova:** Investigation. **E.I. Shagimardanova:** Investigation. **A.M. Rogov:** Investigation, Visualization. **T.R. Tagirova:** Resources, Investigation. **Z.G. Gimadeev:** Methodology. **M.R. Sharipova:** Supervision.

## Data Availability

Whole genome annotation of two Pseudomonas aeruginosa uropathogenic isolates (Original data) (NCBI, GenBank) Whole genome annotation of two Pseudomonas aeruginosa uropathogenic isolates (Original data) (NCBI, GenBank)
